# Supplementary material for: Formulations Based on Drug Loaded Aptamer-Conjugated Liposomes as a Viable Strategy for the Topical Treatment of Basal Cell Carcinoma—In Vitro Tests
Source: Pharmaceutics. 2021 Jun 11;13(6):866. doi: 10.3390/pharmaceutics13060866 (PMC8231244; doi:10.3390/pharmaceutics13060866)
Supplement: Supplementary file 1 [file pharmaceutics-13-00866-s001.zip › pharmaceutics-1237130-supplementary.pdf]

pharmaceutics

MDPI

Supplementary Materials: Formulations Based on Drug Loaded Aptamer-Conjugated Liposomes as a Viable Strategy for the Topical Treatment of Basal Cell Carcinoma—In Vitro Tests

Anca N. Cadinoiu, Delia M. Rata, Leonard I. Atanase, Cosmin T. Mihai, Simona E. Bacaita and Marcel Popa

1. In Vitro Evaluation of Topical Formulations Biocompatibility with Blood Components

Positive (100% lysis) and negative (0% lysis) control samples were obtained by adding equal volumes of Triton X-100 at a concentration of 2% and normal saline solution, respectively, to RBC suspension. The samples were left in the oven at 37°C for 90, 180 and 300 min. Once every 30 minutes, the samples were gently shaken. After the incubation time, the samples were centrifuged at 2000 rpm for 5 min and 1.5 mL of supernatant was incubated for 30 min at room temperature to allow hemoglobin oxidation. Oxyhemoglobin absorbance in supernatants was measured using a Nanodrop One UV-Vis Spectrophotometer at 540 nm. Hemolysis percentage of the RBC was calculated using the Equation (1):

$$\%Hemolysis = \frac{(Abs_{sample} - Abs_{negative\ control})}{(Abs_{positive\ control} - Abs_{negative\ control})} * 100 \quad (1)$$

The experiments were performed in triplicate.

2. In Vitro Transdermal Diffusion Assays

Figure S1 presents the 5-Fluorouracil (5-FU) permeation profiles across the artificial membrane Strat-M® from free 5-FU solution and drug-loaded liposomes over a time period of 24 h.

Figure S1 illustrates that the analyzed samples exhibits regular drug permeation during experiment. Drug's incorporation into the liposomes reduced permeation rate of 5-FU across the artificial membrane. Therefore, the drug permeability value was lower for L4 sample (about 3.06 μg/cm^2 of 5-FU) than that observed for 5-FU solution (about 15.36 μg/cm^2 5-FU).

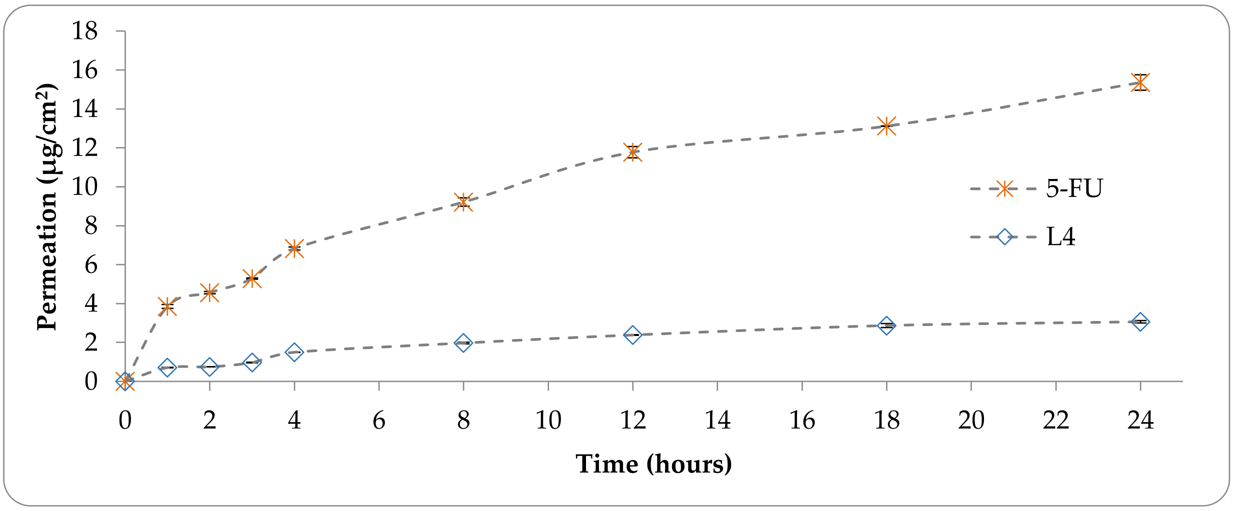

Figure S1. In vitro permeation profiles (μg/cm^2) of 5-FU across Strat-M membrane in phosphate buffer solution (pH 7.4) from free 5-FU solution and drug-loaded liposomes L4.

3. In Vitro Evaluation of topical Formulations Biocompatibility with Blood Components

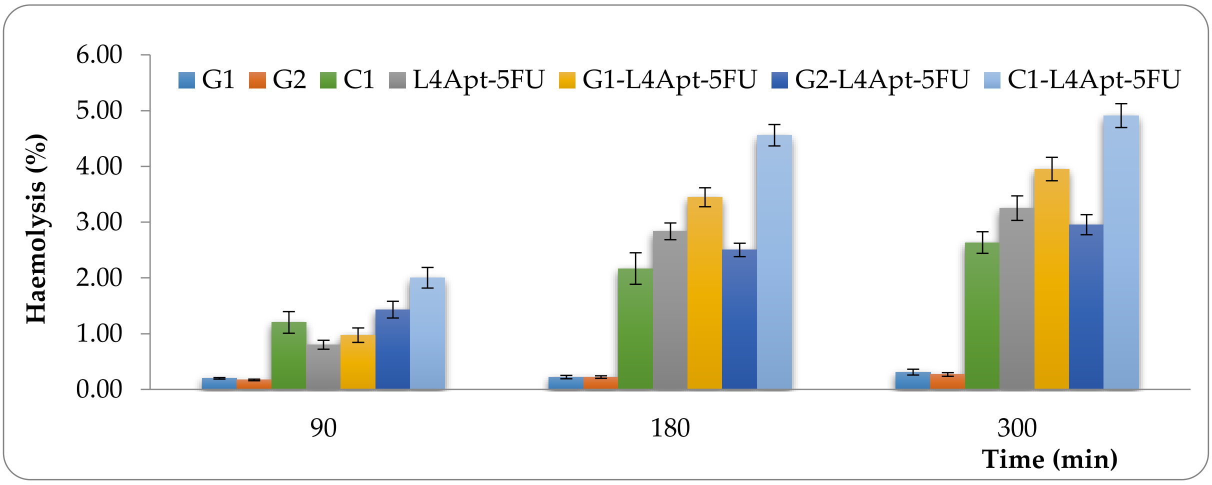

Figure S2. In vitro RBCs lysis of liposome and liposomes formulation loaded with 5-FU.

Where:

Aptamer-conjugated liposomes loaded with 5-FU — L4Apt-5FU

Gel formulations — G1, G2 and C1

Gel formulation containing aptamer-conjugated liposomes loaded with 5-FU —G1–L4Apt-5FU, G2– L4Apt-5FU and C1– L4Apt-5FU

4. Cell Viability Assessment by the MTT Method

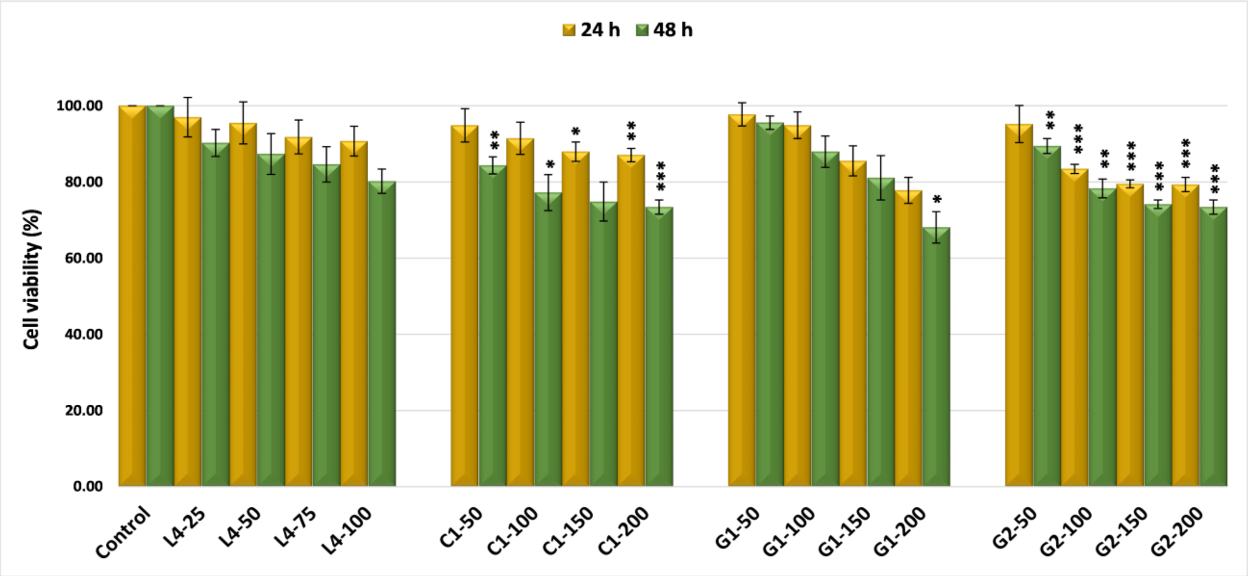

Figure S3. Effect of 24 and 48 h treatment, with different concentrations (μg/mL) of L4, C1, G1, and G2 formulations on the viability of TE 354.T tumor cell cultures (significance different from control: * p <0.05, ** p <0.01, and *** p <0.001).
